# Establishing the bidirectional relationship between depression and subclinical arteriosclerosis – rationale, design, and characteristics of the BiDirect Study

**DOI:** 10.1186/1471-244X-14-174

**Published:** 2014-06-13

**Authors:** Henning Teismann, Heike Wersching, Maren Nagel, Volker Arolt, Walter Heindel, Bernhard T Baune, Jürgen Wellmann, Hans-Werner Hense, Klaus Berger

**Affiliations:** 1 Institute of Epidemiology and Social Medicine, University of Münster, Münster, Germany; 2 Department for Psychiatry and Psychotherapy, University of Münster, Münster, Germany; 3 Department for Clinical Radiology, University of Münster, Münster, Germany; 4 Discipline of Psychiatry, University of Adelaide, Adelaide, Australia

**Keywords:** Depression, Depression subtypes, Arteriosclerosis, Cardiovascular, Cerebrovascular, (f)MRI, White matter hyperintensities, Prospective cohort study, Bidirectional

## Abstract

**Background:**

Depression and cardiovascular diseases due to arteriosclerosis are both frequent and impairing conditions. Depression and (subclinical) arteriosclerosis appear to be related in a bidirectional way, and it is plausible to assume a partly joint causal relationship. However, the biological mechanisms and the behavioral pathways that lead from depression to arteriosclerosis and vice versa remain to be exactly determined.

**Methods/design:**

This study protocol describes the rationale and design of the prospective BiDirect Study that aims at investigating the mutual relationship between depression and (subclinical) arteriosclerosis. BiDirect is scheduled to follow-up three distinct cohorts of individuals ((i) patients with acute depression (N = 999), (ii) patients after an acute cardiac event (N = 347), and (iii) reference subjects from the general population (N = 912)). Over the course of 12 years, four personal examinations are planned to be conducted. The core examination program, which will remain identical across follow-ups, comprises a personal interview (e.g. medical diagnoses, health care utilization, lifestyle and risk behavior), a battery of self-administered questionnaires (e.g. depressive symptoms, readiness to change health behavior, perceived health-related quality of life), sensory (e.g. olfaction, pain) and neuropsychological (e.g. memory, executive functions, emotional processing, manual dexterity) assessments, anthropometry, body impedance measurement, a clinical work-up regarding the vascular status (e.g. electrocardiogram, blood pressure, intima media thickness), the taking of blood samples (serum and plasma, DNA), and structural and functional resonance imaging of the brain (e.g. diffusion tensor imaging, resting-state, emotional faces processing). The present report includes BiDirect-Baseline, the first data collection wave.

**Discussion:**

Due to its prospective character, the integration of three distinct cohorts, the long follow-up time window, the diligent diagnosis of depression taking depression subtypes into account, the consideration of relevant comorbidities and risk factors, the assessment of indicators of (subclinical) arteriosclerosis in different vascular territories, and the structural and functional brain imaging that is performed for a large number of participants, the BiDirect Study represents an innovative approach that combines population-based cohorts with sophisticated clinical work-up methods and that holds the potential to overcome many of the drawbacks characterizing earlier investigations.

## Background

Depression and cardiovascular diseases (CVD) are prevalent disorders in the general population. Both conditions have a major impact on the affected individuals and, in terms of quality of care and costs, on the health care system [1]. Unipolar depressive disorders and ischemic heart diseases are expected to become the two most common causes of disability worldwide by 2030 [2].

Depression is a systemic and rather multifaceted and heterogeneous illness. However, the term depression is used for a variety of phenomena. Due to the non-specificity of diagnostic categories (i.e. ICD-10, DSM-IV-TR) and the response of depression to various treatment modalities (i.e. medication, psychotherapy), the attempt to form biologically meaningful subgroups of depressed patients, so-called subtypes, has recently attracted considerable attention [3]. The concept of depression subtypes - i.e. atypical, melancholic, bipolar, or dysthymic depression - postulates that each may relate to the risk of developing CVD in a specific way [4].

CVDs, in turn, are a group of disorders of the heart and blood vessels that include (amongst others) coronary heart disease, cerebrovascular disease, and peripheral arterial disease. According to the definition of the World Health Organization, CVDs due to arteriosclerosis are among the leading causes of morbidity and mortality in Western countries. Arteriosclerosis is a complex systemic condition that simultaneously affects large and small arteries in different vascular territories. Arteriosclerotic lesions can develop early in life; however, the disease usually progresses slowly and remains subclinical until sudden clinical events such as myocardial infarction or stroke occur [5,6]. The presence of lesions in the white matter of the brain (so-called white matter hyperintensities), which can be detected with brain imaging methods such as magnetic resonance imaging (MRI), can be an indicator of *cerebral* arteriosclerosis [7]. Although not routinely considered, measurements of (subclinical) arteriosclerosis appear particularly suited to study the association between depression and CVD: in contrast to counting clinical events like myocardial infarction or stroke, the degree of arteriosclerosis is a continuous outcome variable that can be operationalized via several different measures, and that can be collected for every study participant with comparatively little effort and burden.

Arteriosclerosis and depression appear to be related, and the current literature suggests that this relationship is bidirectional [1]. First, depression has shown to be both a robust and significant risk and prognostic marker for CVD incidence [8] and an independent predictor of poorer CVD outcome [4,9,10]. Next, a considerable proportion of CVD patients develop depression, leading to significantly increased morbidity and mortality [11]. Finally, patients suffering from both CVD and depression at the same time have a significantly increased risk for future cardiac events compared to CVD patients without depression [4].

Given the apparent association between depression and CVD, it appears plausible to assume a partly joint causal relationship. Studies addressing this issue have mainly focused on potentially shared biological mechanisms. They found that the autonomic nervous system, platelet receptors and function, coagulation factors (e.g. fibrinogen, PAI-1), pro-inflammatory cytokines, endothelial function as well as hormonal and genetic processes may be involved [1,4,12]. In addition to shared biological determinants, there are behavioral and cognitive pathways that may lead from depression to CVD and vice versa. Thus, specific behavioral and/or lifestyle factors that are frequently observed in patients with depression (e.g. smoking, obesity, inactivity, poor diet, poor treatment adherence) may modify the risk to develop subsequent CVD [13,14]. In particular, physical inactivity seems to play an important role [15,16]. Crucially, there is evidence indicating that patients suffering from certain subtypes of depression (e.g. patients with atypical depression) are prone to show such maladaptive behavior, emphasizing the need to establish a classification of depression that takes different subtypes into account. In addition, it appears reasonable to consider known and prognostically important clinical characteristics of depression, such as the number of depressive episodes, symptom severity, or age at first onset and disease duration. These factors have received little attention in the published literature so far. Patients with CVD may perceive themselves as being confronted with actual or imminent burdens (e.g. heart symptoms, changes in cognitive functioning) and losses (e.g. reduced quality of life, decrease in independence or functional capacity), which could contribute to the increased risk of developing subsequent depression. Again, it appears worthwhile to focus closely on the actual manifestation of depression, as certain subtypes or features may appear more frequently than others. Certain demographic factors such as age, gender, socio-economic status, or psychosocial factors (e.g. personality, social integration, adverse life events) and comorbidities such as history of depression, anxiety, or childhood trauma might modify the risk to develop either depression, CVD, or one of the two diseases subsequent to the other one.

Noteworthy, due to methodological aspects of previous studies, there is an ongoing debate as to whether depression can be safely considered as an independent risk factor for CVD. Among the shortcomings were cross-sectional designs, short follow-up periods, exclusive reliance on depression scores for the classification of depression, non-accountancy of somatic comorbidities or classical cardiovascular risk factors, non-consideration of the interaction between age and gender and depression and CVD, and delayed retrospective inquiry of depressive symptoms potentially preceding the cardiac event [17].

In the present article, we describe the goals and design of the BiDirect Study [18], which investigates the mutual relationship between depression and (subclinical) arteriosclerosis. Due to the prospective character of the study, the integration of three distinct cohorts (i.e. patients with depression, patients with CVD, and reference subjects from the same general population), the scheduled follow-up time window of 12 years, the diligent diagnosis of depression, the assessment of relevant comorbidities and risk factors, and the focus on potential age and gender effects, BiDirect holds the potential to overcome many of the drawbacks characterizing earlier investigations.

This report includes the first data collection wave (BiDirect-Baseline), which was conducted from July 2010 to June 2013. The BiDirect Study is scheduled to end after the third follow-up which is planned to be complete in summer 2020.

## Methods/design

### Objectives

The primary objectives of BiDirect are to investigate (i) whether depression contributes to the onset and severity of (subclinical) arteriosclerosis, and (ii) whether the severity of (subclinical) arteriosclerosis is related to incident depression. The focus will be on the course of depression, depression subtypes, lifestyle-related behavioral risk factors, and gender. In detail, the following hypotheses will be evaluated: (1) Patients with depression have more (subclinical) arteriosclerosis than non-depressed individuals of the same age and gender. The severity of (subclinical) arteriosclerosis is related to the prior course of depression and differs between subtypes. (2) Patients with depression show a more unfavorable lifestyle compared to non-depressed individuals. (3) Patients with CVD are at increased risk to develop depression compared to individuals without CVD and present with different subtypes of depression rather than merely adjustment disorder with depressed mood. (4) Patients with *both* CVD and depression show a more unfavorable lifestyle and present with prevailing physical symptoms of depression compared to patients with solely CVD. (5) Gender modifies the relationship between depression and (subclinical) arteriosclerosis. (6) The severity of (subclinical) arteriosclerosis and the subtype of depression allow describing pathophysiological pathways that are shared by both diseases.

### Study design

BiDirect is a prospective observational study that integrates three different cohorts. In addition to BiDirect-Baseline, three follow-up examinations are scheduled to be conducted over the course of 12 years. While the core examination program will remain unchanged across follow-ups, modifications (i.e. expansions and reductions) due to changing demands are allowed for. A short questionnaire is mailed to all participants one year after each of these examinations. This questionnaire covers the perceived health state (and recent changes thereof), recent medical diagnoses, work and living situation, new life events, perceived depressive symptoms, and current medication.

### Recruitment of participants

The study was approved by the ethics committee of the University of Münster and the Westphalian Chamber of Physicians in Münster, North-Rhine-Westphalia, Germany. Written informed consent for participation in the study was obtained from all participants. Overall, 2315 participants were recruited for BiDirect-Baseline; 2258 (97.5%) of them matched the final inclusion criteria. Basic demographic characteristics of the three cohorts are summarized in Table 1.

**Table 1:** Basic demographic characteristics (absolute and relative frequencies) of the BiDirect-Baseline participants stratified by cohort

	**Cohort**		
**Variable**	**Depression (N = 999)**	**CVD (N = 347)**	**Reference (N = 912)**
Sex			
Female	593 (59.4%)	49 (14.1%)	464 (50.9%)
Male	406 (40.6%)	298 (85.9%)	448 (49.1%)
Age			
35 to 44 years	285 (28.5%)	29 (8.4%)	194 (21.3%)
45 to 54 years	437 (43.7%)	128 (36.9%)	304 (33.3%)
55 to 65 years	277 (27.7%)	190 (54.8%)	414 (45.4%)
Educational level			
University degree	238 (23.8%)	99 (28.5%)	375 (41.1%)
University entrance diploma or vocation diploma	181 (18.1%)	56 (16.1%)	157 (17.2%)
General certificate of secondary education	278 (27.8%)	86 (24.8%)	187 (20.5%)
Certificate of secondary education or lower	295 (29.5%)	104 (30.0%)	189 (20.7%)
Net household income^1^ per month			
≤ 2000 Euro	345 (34.5%)	56 (16.1%)	182 (20.0%)
> 2000 Euro	651 (65.2%)	290 (83.6%)	726 (79.6%)

^1^Sum across all household members after tax and social charges.

Cohort 1 consisted of 999 patients, who suffered from an episode of depression at the time of recruitment. Recruitment took place at six different psychiatric and psychosomatic hospitals and departments located in and around the city of Münster (radius: 35 km), as well as two resident psychiatrists’ practices located in Münster. The recruitment of outpatients was limited to those who had been hospitalized due to depression at least once during the 12 months period prior to inclusion into the study. Inclusion criteria were (i) age (≥35 and < 66 years) and (ii) current in- or outpatient treatment due to acute depression. Exclusion criteria were (i) compulsory admission, (ii) comorbid dementia, and (iii) comorbid drug abuse (including alcohol). Potential participants were ascertained by trained and certified study psychologists, and eligible patients were invited to participate in BiDirect-Baseline. Appointments were scheduled via telephone or email.

Cohort 2 comprised 347 patients with CVD (excluding cerebrovascular disease) who were recruited by trained and certified study nurses in four different cardiology departments and rehabilitation clinics in and around Münster (radius: 50 km). Inclusion criteria were (i) age (≥35 and < 66 years) and (ii) acute myocardial infarction, or acute coronary syndrome requiring therapy, or treatment of cardiac disease due to myocardial infarction within the last three months. In case of consent, the CVD patients were invited to participate in BiDirect-Baseline two months after recruitment by the study nurse, and appointments were scheduled via telephone or email.

Cohort 3 (reference cohort) included 912 community-dwelling adults (age: ≥ 35 and < 66 years). The participants had been randomly sampled from the population register of the city of Münster and were invited for BiDirect-Baseline via letter; appointments were scheduled by telephone or email.

Baseline response rates for the depression and CVD cohorts could not be estimated. In both cases, recruitment took place on site, and potential participants could be addressed by several different physicians or members of the nursing staff; therefore, the basic populations of those who were asked to participate are not known. The baseline response rate in the reference cohort was 41.5%.

### Diagnosis of depression

The thorough assessment of depressive symptoms is a key concern of BiDirect-Baseline and a prerequisite for the testing of the specific BiDirect hypotheses over the course of the study. During BiDirect-Baseline, different measures were combined in order to confirm psychiatric diagnoses and to determine whether a participant suffered from acute depression with melancholic or atypical features. For patients from cohort 1, assessment of depression was conducted during recruitment. For participants from cohorts 2 and 3, (when necessary) diagnosis of depression took place during the BiDirect-Baseline examination at the study center.

For diagnostic purposes, all participants received selected modules (i.e. modules A, A’, B, D, and O) of the M.I.N.I. International Neuropsychiatric Interview (German version 5.0.0) [19], which assessed whether a participant exhibited acute (first or recurrent) major depression with or without melancholic features, acute dysthymia, acute/former manic/hypomanic episodes, or acute generalized anxiety disorder. Further, for all patients from cohort 1 and for those participants from cohorts 2 and 3 who had received a M.I.N.I. diagnosis of acute major depression, it was clarified whether atypical depression features were currently present by means of six selected items of the Inventory of Depressive Symptomatology (IDS) [20] (items 8, 12, 14, 27, 29, and 30). In addition, all patients with depression were assessed using the 17 items version of the Hamilton Depression Rating Scale (HAM-D-17) [21] and the 14 items version of the Hamilton Anxiety Rating Scale (HAM-A-14) [22]. It was also assessed whether a participant ever had episodes of melancholic or atypical depression throughout lifetime. Eventually, for explorative purposes we formed so-called “probable” subtypes: if a participant showed all but one criterion for melancholic or atypical depression, the diagnoses “probable melancholic depression” or “probable atypical depression” were assigned.

Notably, in some cases melancholic and atypical features of depression were present at the same time (subtype mixed). In other cases, an assignment to one of the categories melancholic depression or atypical depression was not possible (subtype undifferentiated). Table 2 summarizes the frequencies of the different depression subtypes in cohort 1.

**Table 2:** **Absolute and relative frequencies of depression subtypes among N = 920**^**1 **^**individuals from the depression cohort**

**Subtype of depression (N = 920**^**1**^**)**	**Frequency (percentage)**
Melancholic	554 (60.2%)
Atypical	44 (4.8%)
Mixed	95 (10.3%)
Probably melancholic	27 (2.9%)
Probably atypical	39 (4.2%)
Probably mixed	68 (7.4%)
Melancholic + probably melancholic	581 (63.2%)
Atypical + probably atypical	83 (9.0%)
Mixed + probably mixed	163 (17.7%)
Undifferentiated	93 (10.1%)

^1^Number of members of the depression cohort (N = 999) for whom the subtype diagnostic algorithm could be successfully completed.

### Methods against bias

Selection bias was reduced by cooperation with the hospitals providing in- or outpatient treatment for psychiatric and cardiologic diseases to the population of the city of Münster. Thus, newly occurring cases of depression, myocardial infarction, and acute coronary syndrome requiring in-hospital treatment in the participating institutions during the critical period fulfilling inclusion criteria and willing to participate could be recruited. The incorporation of a population-based reference cohort is a prerequisite for the analysis of the association between depression and (subclinical) arteriosclerosis in a truly bidirectional way. The reference subjects were randomly sampled from the population (i.e. inhabitants of the city of Münster) that gave rise to the depression and CVD cohorts. Moreover, we assessed the majority of variables known to be potential confounders of the association between depression and CVD. Among them are socio-demographic factors, existing comorbidities, lifestyle factors, and current medications. The assessment of these variables allows to control their effects in the applied statistical models and to evaluate their roles as either confounders or mediators.

### Data collection procedures

The large majority of the data for BiDirect-Baseline (with the exception of the psychiatric interviews, which were administered by trained and certified study psychologists in most cases at the hospitals where recruitment took place) was collected by trained and certified study nurses at the BiDirect study center, which is an integral part of the Institute for Epidemiology and Social Medicine, University of Münster, Germany. The MRI scans were obtained by the staff of the Department for Clinical Radiology, University of Münster.

### Constructs and instruments

The BiDirect-Baseline examination included several different modules: (i) computer assisted personal interview (CAPI), (ii) psychiatric interview (where required), (iii) self-administered questionnaires, (iv) assessment of sensory/cognitive functioning, and (v) diagnostic work-up (including MRI of the brain and taking of blood samples). Completing the entire program required an average of three-and-a-half hours.

By means of the *CAPI*, which was applied in German language, detailed information regarding socio-demographic and socio-economic background, lifetime medical diagnoses, health care utilization behavior, insurance status, lifestyle and risk behavior (i.e. diet, physical activity, alcohol consumption, smoking), and current medication was obtained, for the most part using standardized sets of closed questions which had previously been used in different studies [23,24]. In addition, there were sections devoted to perceived health state. In case of female participants, the number of children, oral contraceptive usage, and certain surgical interventions (i.e. hysterectomy, ovariectomy) were inquired. Notably, for the participants of cohorts 2 and 3, the diagnostic M.I.N.I. interview was included in the CAPI. If indicated by the M.I.N.I. results, an additional appointment was scheduled, where additional non-structured and structured (HAM-D-17, HAM-A-14, selected IDS items) psychiatric interviews were administered by trained and certified study psychologists. The goal of these interviews was to assess the severity of depression and anxiety symptoms, and to evaluate whether or not a participant suffered from melancholic or atypical depression. For patients from cohort 1, the diagnostic and psychiatric interviews had already been conducted during recruitment (cf. section “Diagnosis of depression”).

Several different *self-administered questionnaires* or sets of questions, which were combined to form a single booklet, were completed by the participants. The following instruments/sets were used in given order: (i) the Pittsburgh Sleep Quality Index (PSQI) [25], which assesses sleep quality and disturbances over a one-month time interval; (ii) the Center for Epidemiological Studies Depression Scale (CES-D) [26], which was designed to measure depressive symptomatology over a one-week time interval; (iii) a headache questionnaire [27]; (iv) the Pain Sensitivity Questionnaire (PSQ) [28], a measure of pain perception based on imagined painful daily life situations; (v) a set of six questions regarding perceived memory functioning and recent changes thereof; (vi) the Childhood Trauma Screener (CTS) [29], which covers general living conditions and traumata during childhood (performed in a subsample of the baseline participants); (vii) a set of seven questions assessing the readiness to change health behavior (i.e. physical activity, diet, weight, alcohol consumption, smoking) according to the transtheoretical model [30]; and (viii) the EQ-5D [31], which assesses the perceived health-related quality of life.

In order to test *sensory functioning*, an orienting assessment of color perception was performed using 14 Ishihara Color Plates. Further, visual acuity was assessed (left vs. right eye). Moreover, odor perception was examined using the Sniffin’ Sticks Screening 12 Test (Burghart Messtechnik GmbH, Wedel, Germany), a forced choice test which allows categorizing participants into anosmic, hyposmic, and normosmic based on 12 different odors. Further, the pressure pain threshold was determined using a pain test algometer (Force Dial FDK/FDN Force Gage, Wagner Instruments, Greenwich, CT, USA) for left and right index fingers separately.

The *cognitive functioning* module included the assessment of cognitive processing speed, verbal working and long-term memory, executive functions (verbal fluency, interference disposition, flexibility), emotional processing, and manual dexterity. The following tests were administered in given order: (i) immediate recall of a sequence of 12 (recorded and replayed) spoken German nouns, each with an emotional load (positive, neutral, or negative) (cf. [32]); (ii) verbal production of as many different animal names as possible within a time interval of one minute [33]; (iii) Color-Word-Interference Test [34]; (iv) Trail Making Tests A and B [35]; (v) delayed recall of the nouns (see above); (vi) rating of the nouns regarding valence and arousal on nine-point rating scales; and (vii) Purdue Pegboard Test [36] for left and right hands separately. Eventually, the participants self-evaluated their level of attention during the cognitive functioning module on an eleven-point rating scale.

The diagnostic work-up began with the determination of weight (without shoes and heavier clothes), height, and waist circumference. Bioelectrical impedance measurement (Body Impedance Analyzer BIA 2000-S, Data Input GmbH) including the determination of body fat and water, extracellular mass, body cell mass, and basic metabolic rate was applied. The determination of the vascular status consisted of the measurement of a standard 3-channel electrocardiogram (ECG) using extremity leads, the brachial systolic and diastolic blood pressure by oscillometry, the ankle-brachial index by photoplethysmography, the pulse wave velocity, the augmentation index (Vascular Explorer, enverdis GmbH), and the intima-media-thickness of the far wall of the carotid arteries non-invasively by ultrasound (Acuson X300, Siemens). In addition, blood samples were taken (plasma, serum, and DNA), and fundus photography (Digital Retinography System, EyeNovation) was performed in a subsample of the baseline participants. The diagnostic-workup closed with an MRI of the brain, which involved both structural (3D-T1, T2*, FLAIR, and DTI sequences) and functional (resting-state sequence, emotional faces paradigm [37]) recordings. Table 3 gives an overview regarding the number of completed examinations per cohort.

**Table 3:** **Absolute and relative frequencies of examinations completed**^**1 **^**by the BiDirect-Baseline participants stratified by cohort**

	**Cohort**		
**Examination**	**Depression (N = 999)**	**CVD (N = 347)**	**Reference (N = 912)**
Computer-assisted personal interview	996 (99.7%)	346 (99.7%)	911 (99.9%)
Sensory functioning			
Pain	953 (95.4%)	304 (87.6%)	795 (87.2%)
Olfaction	989 (99.0%)	341 (98.3%)	901 (98.8%)
Visual acuity	996 (99.7%)	344 (99.1%)	907 (99.5%)
Cognitive functioning	964 (95.5%)	332 (95.7%)	824 (90.4%)
Anthropometry	998 (99.9%)	347 (100.0%)	890 (97.6%)
Body impedance	984 (98.5%)	331 (95.4%)	896 (98.2%)
Vascular status			
Electrocardiogram	988 (98.9%)	345 (99.4%)	905 (99.2%)
Blood pressure	987 (98.8%)	340 (98.0%)	902 (98.9%)
Intima media thickness	987 (98.8%)	345 (99.4%)	894 (98.0%)
Blood sample			
DNA	929 (93.0%)	321 (92.5%)	783 (85.9%)
Serum and plasma	895 (89.6%)	302 (87.0%)	742 (81.4%)
MRI			
Anatomical	735 (73.6%)	52 (15.0%)	669 (73.4%)
Resting state	719 (72.0%)	51 (14.7%)	654 (71.7%)
Emotional faces	621 (62.2%)	43 (12.4%)	528 (57.9%)

^1^No refusal by participant or abortion by participant/examiner.

### Quality assurance and data management

The primary goal of all quality assurance measures was to generate high quality data. In principle, we followed the rules for good epidemiological practice as well as the recommendations for the use and storage of blood samples and DNA as provided by the German Society of Epidemiology (cf. http://dgepi.de/fileadmin/pdf/leitlinien/GEP_LL_english_f.pdf). The storage of biomaterials was split between different refrigerators, all of which were monitored by a central alert system 24 hours per day seven days per week. The BiDirect standard operations manual contains operating procedures for all BiDirect interview and examination components.

Prior to the beginning of BiDirect-Baseline, the members of the study team (i.e. study nurses, study psychologists) underwent an initial three-months training ending with certifications. Moreover, each member of the study staff was trained and certified for a variety of tasks, providing mutual backup in case of illness or changes in personnel. Performance was closely monitored and routinely assessed.

Data management has been carried out in parallel with data collection, based on standardized and partly automated procedures for data processing and plausibility checking. Data backup routines are scheduled on a daily basis.

### Biometric concept and statistical analyses

The primary endpoints of BiDirect are incidence and/or progression of both depressive symptoms and (subclinical) arteriosclerosis. Secondary endpoints are onset of depression, myocardial infarction, brain infarction, and death. Current data evaluation is restricted to the baseline cross-sectional analyses, since the first follow-up period is still ongoing. Depending on the outcome scale level (continuous vs. categorical) and time point of assessment (baseline vs. follow-up), the statistical methods comprise multivariable linear or logistic regression analyses, accounting for time-varying predictors and repeated outcome assessment.

The primary endpoints are operationalized by closed questions and non-invasive clinical examinations. The majority of primary endpoints yield continuous variables (e.g. CES-D summary score, systolic/diastolic blood pressure [mmHg], intima media thickness [mm]) with change over time. The computation of arteriosclerosis scores will facilitate the analysis of disease progression in different vascular territories. Power calculations were based on the planned study duration of 12 years and took secondary, frequency-type endpoints (in particular subclinical brain pathology on the MRI as the limiting factor) into account. Loss-to-follow-up between subsequent examinations was expected to be maximally 20% on average across cohorts.

## Discussion

BiDirect-Baseline, the first data collection wave of the longitudinal BiDirect Study, has been successfully completed, and forms the foundation for investigating the bidirectional relationship between depression and (subclinical) arteriosclerosis prospectively.

The burden of depression and CVD on the health care system is continuously growing. Therefore, it is significant to state that a number of aspects of BiDirect are innovative and can be expected to generate knowledge regarding the underlying biological (i.e. inflammatory, hormonal) and behavioral (i.e. lifestyle-related) mechanisms that may lead to more specific and more effective treatment strategies in the future.

First and foremost, BiDirect takes a distinguished view on depression. While many previous studies investigating the relationship between depression and CVD adopted a non-differential, single-diagnostic-entity concept of depression, BiDirect will explicitly discriminate between different subtypes. The magnitude of the association between depression and CVD appears to vary by depression subtype, and thus it is possible that subtype-specific biological mechanisms exist that link the two conditions. A literature review of our group [12] came to the conclusion that there are indications for subtype-specific associations with regard to at least immune activation and hypothalamic-pituitary-adrenal axis hyperactivity. However, so far there are no prospective studies addressing this issue, and BiDirect will be in the first line to closing this gap. There are different ways to classify patients with depression, among them etiology-based, treatment-based, time-of-onset-based, and gender-based approaches [3]. The most established way is to form subtypes due to prevalent constellations of symptoms. Our present classification based on M.I.N.I. and IDS results is one such example. However, we plan to apply further, more data-driven strategies to differentiate patients with different forms of depression. For instance, it may be possible to classify patients based on the available biologic and genetic markers.

Secondly, BiDirect includes the assessment of indicators of (subclinical) arteriosclerosis in different vascular territories. This enables, in addition to event-type endpoints of clinical manifestation (i.e. myocardial infarction, stroke, death), the assessment of further (continuous) outcomes (e.g. ankle-brachial blood pressure index, pulse-wave velocity, intima-media-thickness). The differentiation between arteriosclerosis endpoints, similar to the discrimination between depression subtypes, is expected to contribute to a better understanding of the biological and behavioral mechanisms underlying the depression – CVD relationship. Further, combinations of vascular parameters over different vascular territories, e.g. in the form of arteriosclerosis scores, may help to better elucidate the subclinical arteriosclerotic burden and, in particular, its change over time.

Finally, structural and functional brain imaging is performed for a large number of participants. The *structural* MRI data display (pathological) anatomical changes, which can be quantified, for instance, by means of voxel-based morphometry. Of primary interest in BiDirect are so-called white-matter hyperintensities, which are considered to be potential precursors of e.g. stroke and vascular depression [38-40]. The *functional* MRI data allow analyses of both the activation and connectivity of brain regions. Using a facial expression processing task [37], the activation of the cortico-limbic circuitry involved in emotion processing and regulation can be studied. Of particular interest in BiDirect are amygdala, dorsolateral prefrontal cortex, dorsal anterior cingulate cortex, and lateral orbitofrontal cortex. The task-free resting-state fMRI sequence allows the evaluation of functional connectivity of distant brain regions and thus of the integrity of large-scale brain networks [41].

In conclusion, the BiDirect Study represents an innovative approach, combining population-based cohorts with sophisticated clinical work-up methods. The BiDirect team welcomes collaborations with both internal and external researchers.

## Competing interests

The authors declare that they have no competing interests.

## Authors’ contributions

HT analyzed the data and drafted the manuscript. HW and MN made substantial contributions to the acquisition and analysis of the data. VA and WH contributed to the conception of the study, provided specific knowledge, and revised the manuscript critically for important intellectual content. BTB and HWH made substantial contributions to the conception and design of the study. JW made substantial contributions to the analysis of the data. KB conceived of the study and supervised its design, coordination, and execution. All authors read and approved the final manuscript.

## Pre-publication history

The pre-publication history for this paper can be accessed here:

http://www.biomedcentral.com/1471-244X/14/174/prepub
